# Molecular Genetics of the Usher Syndrome in Lebanon: Identification of 11 Novel Protein Truncating Mutations by Whole Exome Sequencing

**DOI:** 10.1371/journal.pone.0107326

**Published:** 2014-09-11

**Authors:** Ramesh Reddy, Somayyeh Fahiminiya, Elie El Zir, Ahmad Mansour, Andre Megarbane, Jacek Majewski, Rima Slim

**Affiliations:** 1 Departments of Human Genetics and Obstetrics-Gynecology, McGill University Health Centre, Montreal, Canada; 2 McGill University and Genome Quebec Innovation Centre and Department of Human Genetics, Montreal, Canada; 3 Department of Otorhinolaryngology, Hôpital Sacré-Coeur, Baabda, Lebanon; 4 Department of Ophthalmology, American University of Beirut, Beirut, Lebanon; 5 Unité de génétique médicale, Faculté de médecine, Université Saint Joseph, Beirut, Lebanon; University of Florida, United States of America

## Abstract

**Background:**

Usher syndrome (USH) is a genetically heterogeneous condition with ten disease-causing genes. The spectrum of genes and mutations causing USH in the Lebanese and Middle Eastern populations has not been described. Consequently, diagnostic approaches designed to screen for previously reported mutations were unlikely to identify the mutations in 11 unrelated families, eight of Lebanese and three of Middle Eastern origins. In addition, six of the ten USH genes consist of more than 20 exons, each, which made mutational analysis by Sanger sequencing of PCR-amplified exons from genomic DNA tedious and costly. The study was aimed at the identification of USH causing genes and mutations in 11 unrelated families with USH type I or II.

**Methods:**

Whole exome sequencing followed by expanded familial validation by Sanger sequencing.

**Results:**

We identified disease-causing mutations in all the analyzed patients in four USH genes, *MYO7A*, *USH2A*, *GPR98* and *CDH23*. Eleven of the mutations were novel and protein truncating, including a complex rearrangement in *GPR98*.

**Conclusion:**

Our data highlight the genetic diversity of Usher syndrome in the Lebanese population and the time and cost-effectiveness of whole exome sequencing approach for mutation analysis of genetically heterogeneous conditions caused by large genes.

## Introduction

Usher syndrome (USH) is a group of recessive inherited disorders characterized by neurosensory deafness and progressive vision loss, with or without vestibular dysfunction [1]. USH affects around one in 20,000 individuals and accounts for approximately 50% of all the deaf-blindness cases [2]. Usher syndrome can be clinically classified into three types, USH1, USH2 and USH3 [3]. USH1 is the most severe type of the three with profound congenital deafness, vestibular dysfunction and prepubertal progressive retinitis pigmentosa (RP). USH2 is characterized by moderate to severe congenital deafness and post pubertal onset of RP. USH3 is characterized by progressive hearing loss, retinitis pigmentosa and variable vestibular dysfunction. USH2 is the most common type of USH and accounts for 50–65% of all USH cases [3], [4]. USH1 accounts for 10–35% of all USH cases [3], [4] while USH3 is rare and accounts for 2–5% of USH cases in several populations with the exception of the Finnish and Ashkenazi populations where USH3 accounts for 40% of all USH cases [3], [4].

To date, there are 10 genes known to cause USH (http://hereditaryhearingloss.org). Mutations in 6 genes, *MYO7A* [5], *USH1C* [6], *CDH23* [7], *PCDH15* [8], *USH1G* [9], and *CIB2* [10] have been found causative of USH1. *MYO7A* is the major gene responsible for USH1 and mutations in *MYO7A* alone account for 70% of USH1. *CDH23* is the second commonly mutated gene and mutations in *MYO7A* and *CDH23* together account for 80% of USH1 [11], [12]. Mutations in three genes, *USH2A* [*13*], *GPR98* [*14*], and *DFNB31/WHRN* [*15*] have been identified as disease-causing for USH2. *USH2A* is the most frequently mutated gene in USH2 patients and mutations in *USH2A* alone account for 85% of USH2 while mutations in *GPR98* account for 6% of USH2 in the French and other Caucasian populations [16], [17]. Mutations in one gene *CLRN1* are found causative for USH3 [18]. Digenic inheritance in USH has been reported with mutations in *GPR98* and *PDZD7* [19] in USH2 patients and with mutations in *CDH23* and *PCDH15* [*20*] or *MYO7A* and *PCDH15* [*21*] in USH1 patients. In addition, mutations in *MYO7A, USH1C, DFNB31, CIB2, CDH23* and *PCDH15* were also reported in non-syndromic deafness without retinal degeneration [22], [23] and mutations in *USH2A* and *CLRN1* have been reported in patients with isolated RP or retinal dystrophies and no deafness [24]–[26].

The spectrum of genes and mutations that cause Usher syndrome in the Lebanese and Middle Eastern populations is not known. To date, only two Lebanese USH families, one with a mutation in *USH1C* and one with a mutation in *CLRN1*, have been reported [6], [27]. In this study, we describe the use of whole exome sequencing (WES) to identify Usher causing mutations in 11 unrelated families from Lebanon and the Middle East, of which one was linked to USH1B, three linked to USH2A, and two had weak linkage, with lod score of less than 2, to USH2A in a previous study performed before the identification of the USH1B and USH2A associated genes [28], [29]. Because some of the families did not have a conclusive linkage to known loci and the genetic causes of Usher syndrome in these populations have not been well studied, we favored the use of WES instead of a targeted sequencing approach. We report the identification of 11 novel protein-truncating mutations in four Usher genes in the 11 families. Our data demonstrate the advantage of WES over traditional approaches for DNA diagnosis of genetically heterogeneous disorders caused by mutations in large genes.

## Materials and Methods

### Patient Recruitment

**Figure 1:**
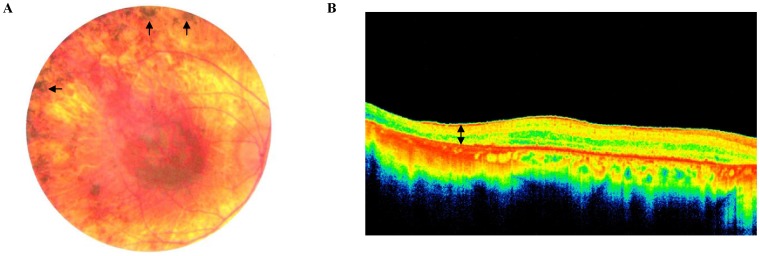
Clinical examination. **A.** Posterior pole of the right eye of patient USHLB13-II.3 showing atrophy of the retina and choroid with pigment spicules (arrows) anterior to the arcades. **B.** Spectral domain optical coherence tomography of the right eye of the same patient showing significant thinning of the retina (space delineated by double arrow) compared to normal controls.

This study adhered to the tenets of the Declaration of Helsinki. The study was approved by the Institutional Review Board of the American University of Beirut. Verbal consents were obtained from all enrolled subjects according to the recommendations of the Institutional Review Board of the American University of Beirut that were in place between 1994–1998 and before the implementation of written consent forms. The verbal consents of the subjects were witnessed by at least three study participants. Patients have been clinically evaluated according to the criteria recommended by the Usher Syndrome Consortium [30]. Comprehensive ophthalmic examinations, including routine eye tests, perimetry and electroretinography (ERG) were carried out for each patient (Figure 1). Auditory function of affected subjects was assessed by pure tone audiometry, speech-audiometry, and tympanometry. Hearing impairment was classified as mild (20–40 dB), moderate (41–70 dB), severe (71–95 dB), or profound (>95 dB). Electronystagmography was used to assess spontaneous and positional nystagmus (saccades and pursuit). A standard caloric test (30°C and 44°C) was performed using an ICS caloric irrigation system and observation of the nystagmus using Frenzel's glasses. Detailed family history was obtained through personal interviews with patients and their relatives (Figures S1 and S2). Peripheral blood samples were collected from all available family members for DNA extraction.

### Clinical classification and linkage analysis

Based on the clinical evaluations of the 11 studied patients, 4 were classified as USH1 and 7 were classified as USH2 (Table 1). Linkage analysis with microsatellite markers surrounding the USH1B locus on 11q13.5 and USH2A on 1q41 were previously reported on six families [28], [29]. Five families were attributed to USH2A and 1 family to USH1B locus.

**Table 1:** Summary of the identified mutations in the four Usher genes.

Family	Ethnicity	Linkage	Proband	Gene	Mutation		Status	Reported
					cDNA	protein		
**Usher Type 1**								
USHEG5	Egyptian	no linkage data	II-1	*MYO7A*	c.2863G>A	p.G955S	Hom	Reported
USHJO9	Jordanian	linked to 11q13.5 [28]	III-2	*MYO7A*	c.462C>A	p.C154*	Hom	Novel
USHLB12	Lebanese	no linkage data	II-2	*MYO7A*	c.834C>A	p.Y278*	Hom	Novel
USHLB14	Lebanese	no linkage data	II-1	*CDH23*	c.8803C>T	p.R2935*	Hom	Novel
**Usher Type 2**								
USHLB1	Lebanese	linked to 1q [29]	III-3	*USH2A*	c.11907delA	p.A3970Lfs*14	Hom	Novel
USHLB6	Lebanese	linked to 1q [29]	II-2	*USH2A*	c.531_532insA	p.T178Nfs*4	Hom	Novel
USHLB8	Lebanese	linked to 1q [29]	II-1	*USH2A*	c.14031_14032insA	p.A4678Sfs*5	Hom	Novel
USHLB13	Lebanese	no linkage data	II-1	*USH2A*	c.4707T>G	p.Y1569*	Het	Novel
				*USH2A*	c.14424C>A	p.C4808*	Het	Novel
USHJO10	Jordanian	no linkage data	III-1	*USH2A*	c.8681G>A	p.R2894K/p.R2853Ifs*5	Hom	Novel
USHLB2	Lebanese	weak linkage	III-2	*GPR98*	c.17756-2239_17856+11702delins17bp	p.G5919Nfs*15	Hom	Novel
USHLB11	Lebanese	weak linkage	II-4	*GPR98*	c.16040delA	p.D5347Vfs*22	Hom	Novel

### Exome capture and sequencing

Exome sequencing was carried out at the McGill University and Genome Quebec Innovation Centre, Montreal, Canada as previously detailed [31], [32]. In brief, whole exome was captured using the SureSelect Human All Exon Kit version 5 (Agilent Technologies, Inc., Santa Clara, CA) on 3 µg of genomic DNA from all the probands of 11 families. Sequencing was carried out with pair end 100 base reads on the Illumina Hiseq2000 sequencer. The reads were aligned to the human reference genome (hg19) using BWA (v. 0.5.9) [33]. The whole exome coverage at 30X resolution in the 11 patient samples was above 95% (data not shown). A mean coverage of 133X was obtained for all consensus coding sequence (CCDS) exons using the Genome Analysis Toolkit (GATK)[34]. Variants were called and annotated using the SAMTools (v. 0.1.17) [35] and ANNOVAR [36], respectively. To remove systematic false positives, as well as common polymorphisms, the lists of variants were filtered against >1000 of individual in-house exomes. Additionally, the variants with an allele frequency >5% in either the 1000 genomes database (http://www.1000genomes.org) or the NHLBI exomes (v.0.0.14, June 20, 2012) were filtered out. Also, we looked for copy number variants (CNVs) in our patient samples using FishingCNV software [37]. Finally, to focus on likely disease-causing variants, we prioritized frameshift, indel, nonsense, missense and canonical splice site variants for further analysis. Additional analyses such as the identification of regions of homozygosity were performed using custom PERL scripts.

### Sanger sequencing validation of identified mutations and bioinformatics analysis

Primers were designed to amplify the fragments containing the mutations using primer 3 software [38]. Sequence analysis was performed using DNASTAR [39] and the sequences were compared to their respective reference sequences (NM_000260.3 for *MYO7A*; NM_206933.2 for *USH2A*; NM_001171933.1 for *CDH23*; and NM_032119.3 for *GPR98*). DNA mutation numbering is based on cDNA sequence with a ‘c.’ symbol before the number and uses the A of the ATG translation initiation start site as nucleotide 1. Protein numbering starts from the initiation codon. Mutations nomenclatures were checked using Mutalyzer [40]. The amino acid and nucleotide residue conservations across species were examined using NCBI BLAST (http://www.ncbi. nlm.nih.gov/BLAST/). Splice site prediction was performed using the following online available prediction tools, NetGene2 (http://www.cbs.dtu.dk/services/NetGene2), HSF (http://www.umd.be/HSF/), BDGP (http://www.fruitfly.org/seq_tools/splice.html), GENSCAN (http://genes.mit.edu/GENSCAN.html). The possible pathogenic effect of protein-coding variants was examined using two prediction tools, SIFT (http://sift.jcvi.org/) and Polyphen-2 (http://genetics.bwh.harvard.edu/pph2/).

## Results

**Figure 2:**
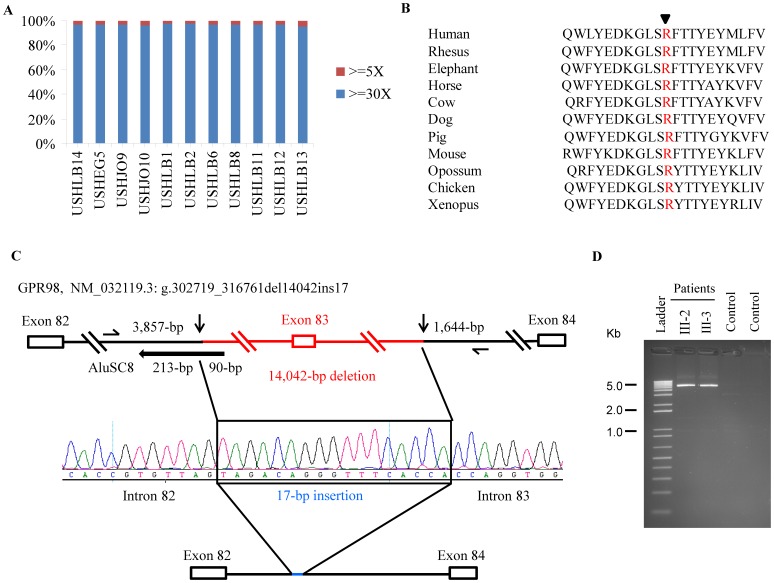
Exome sequencing coverage, conservation of one mutation in *USH2A*, and diagram of the 14,042 bp deletion in *GPR98*. **A.** Image showing the coverage of the reads at >30X and >5X of the 10 Usher genes in the 11 patients studied using whole exome sequencing. **B.** Conservation of the Arginine (R) at amino acid 2894 in USH2A in different species is shown. **C.** Diagram of the rearrangement deleting exon 83 of *GPR98* in family USHLB2. Arrows indicate the proximal and distal breakpoints of the 14,042 bp deletion. Horizontal arrow shows the AluSC8 repeat overlapping the proximal breakpoint. The 17 bp insertion is shown in a rectangle between the flanking sequences. Primers used for amplifying the 5.5 kb fragment are shown. **D.** Shows the 5.5 kb fragment amplified in the two patients III-2 and III-3 from family USHLB2. The fragment was not amplified in the two control samples due to its large size.

WES was performed on one affected member from each of 11 unrelated families. Of these 11 families, four were diagnosed clinically with USH1 and one had linkage to the region containing *MYO7A* [29] (Tables 1 and S1). The remaining 7 families were diagnosed with USH2 and three of them had a conclusive linkage to 1q41 [28] (Tables 1 and S2). The average coverage of the 10 known Usher genes was around 96% in the 11 patients studied (Figure 2A, Table S3). Analysis of the exome sequencing data identified 12 different mutations in (i) two USH1 genes, *MYO7A* and *CDH23*; (ii) two USH2 genes, *USH2A* and *GPR98* (Table 1); and (iii) no mutations in any other USH gene suggesting monogenic inheritance of the disease. Of the 12 identified mutations, three were in *MYO7A*, one in *CDH23*, six in *USH2A*, and two in *GPR98* (Table 1 and Figure S3). One of the 12 mutations was a previously reported missense, p.G955S, in *MYO7A*, and 11 are novel.

The 11 novel mutations included five nonsense in *MYO7A*, *CDH23*, and *USH2A*; four frameshifts in *USH2A* and *GPR98*; a large rearrangement deleting 14,042 bp including exon 83 of *GPR98* and inserting 17 bp; and a missense mutation, R2894K (c.8681 G>A), in *USH2A*. In the latter, the Arginine residue at amino acid position 2894 is highly conserved from Human to Xenopus (Figure 2B) and lies in the Fibronectin-15 domain of the protein and consequently its substitution could have a potential effect on the structure and function of the protein. The effect of this substitution was also predicted to be damaging by polyphen and SIFT prediction programmes [41], [42]. In addition, because this missense is caused by a DNA change affecting the first nucleotide of exon 43 that could potentially affect the gene splicing, we used five *in silico* splice prediction programmes to assess its impact. We found that three programmes, BDGP [43], MaxEntscan [44], and NetGene2 [45], predicted the same abnormal splicing affecting the splice acceptor site of exon 43 of *USH2A* and leading to its skipping from the RNA. This would result in a frameshift and premature protein truncation and is an additional argument in favour of the pathogenicity of this DNA change.

To define the rearrangement around exon 83 of *GPR98* identified by exome sequencing, we used primer walking, regular and long range PCR amplification on DNA from affected and unaffected members of USHLB2. This analysis led us to amplify a 5.5 kb PCR fragment containing the deletion flanking regions (Figure 2C, 2D). DNA sequencing of this fragment revealed that the rearrangement consists of a 14,042 bp deletion and of a 17 bp insertion (Figure 2C). The use of RepeatMasker (www.repeatmasker.org) revealed the presence of an AluSC8 repeat on the proximal breakpoint of the deletion in intron 82 (Figure 2C). Comparing the 17 bp insertion with databases revealed its presence at several genomic loci, but the 17 bp did not map to any known repetitive elements.

All the identified mutations were confirmed in the patients and their extended families using Sanger sequencing and all segregated with the disease phenotype (Tables S2 and S3). The 11 identified novel mutations were not found in the LOVD Usher database (https://grenada.lumc.nl/LOVD2/Usher_montpellier/USHbases.html), the deafness variation database (http://deafnessvariationdatabase.com/), or in any of the control populations listed on the exome variant server database (EVS, http://evs.gs.washington.edu/EVS/), the genome variant database (1000 genomes, http://www.1000genomes.org/), and dbSNP (http://www.ncbi.nlm.nih.gov/snp). Altogether, these arguments demonstrate the pathogenicity of the 12 identified mutations and their causation of USH.

## Discussion

With the advent of WES, finding mutations in genetically heterogeneous Mendelian disorders has become fast and feasible. Recent studies have demonstrated that mutation detection rate using WES in patients with USH1 and USH2 is more than 90% and suggested the usefulness of this approach for routine molecular diagnosis of USH [17], [46]. In our study, we identified the causative genes and mutations in all the 11 families we analyzed, which corroborate previous observations in the field.

**Figure 3:**
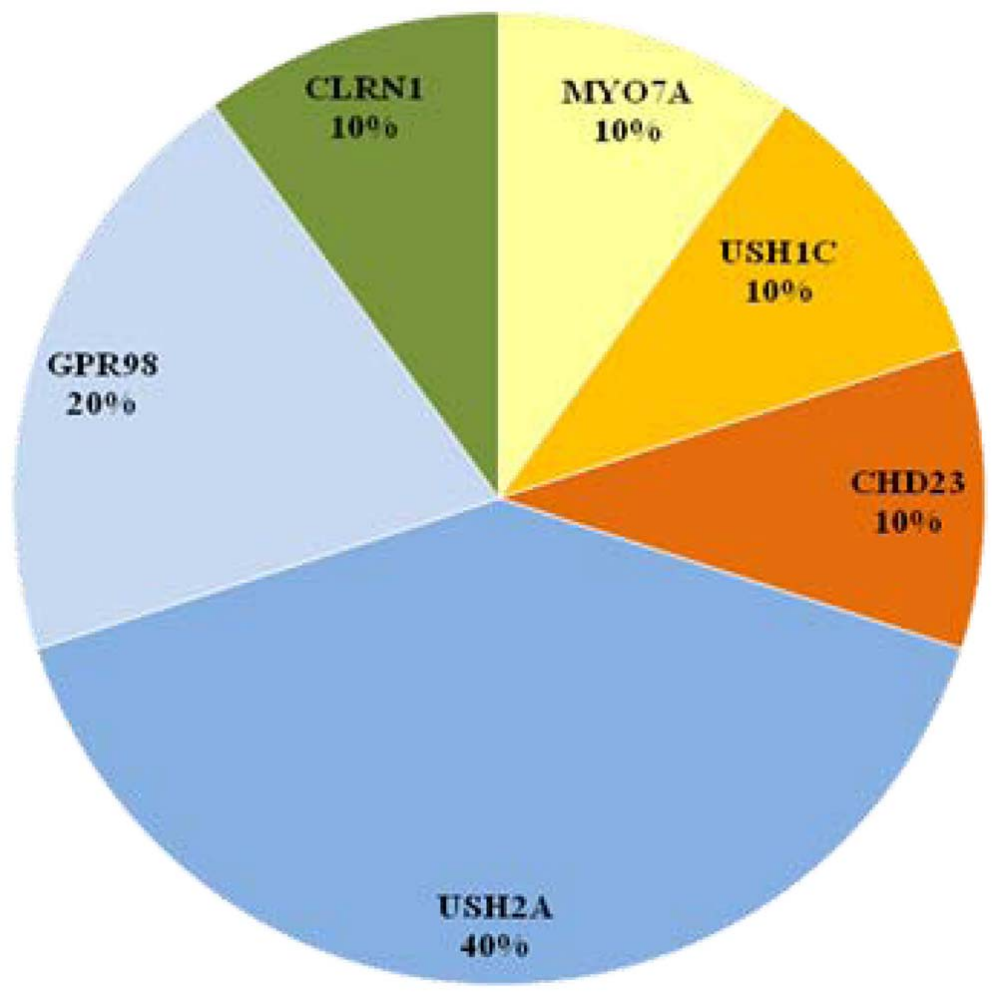
Distribution of mutated genes in ten Lebanese families with USH. Among these ten families, eight are described in this study.

To identify USH genes and mutations in 11 USH families from Lebanon and the Middle East, from which only two families with USH syndrome had been characterized at the mutation level, we performed WES on 11 probands and identified disease-causing mutations in all of them. These patients had 12 different mutations, of which 11 are novel and lead to protein truncations. To date and including this study, mutations in 11 Lebanese families clinically with USH, four with USH1, six with USH2, and one with USH3, have been reported [6], [27]. Among the four USH1 families, four different genes, three responsible for USH1, *USH1C* [6], MYO7A, and *CDH23*, and one responsible for USH3, *CLRN1* [27], were found mutated, one in each family. Among the six USH2 families, two genes, *USH2A* and *GPR98*, were found mutated in four and two families, respectively (Figure 3). The remaining family was originally diagnosed as USH3, but the diagnosis was revised to PHARC (a neurodegenerative disease characterized by polyneuropathy, hearing loss, ataxia, retinitis pigmentosa, and early-onset cataract) after the identification of a homozygous mutation in *ABHD12* and clinical re-evaluation [47]. Our current and previous data on the 11 Lebanese families indicate that *USH2A* mutations (40%) are the most frequent cause of USH in the Lebanese population, followed by *GPR98* mutations (20%) while mutations in all the other USH genes seem to be rarer (Figure 3). Analyzing more families from the Lebanese population will help validating our observation on a larger cohort and drawing stronger conclusions about the spectrum of genes and mutations underlying Usher syndrome in Lebanon.

In *GPR98*, we identified a complex rearrangement resulting in the deletion of a 14,042 bp fragment including exon 83 and the insertion of 17 bp. The presence of an AluSC8 repeat on the proximal side of the deletion indicates a possible non-homologous end joining (NHEJ) at its origin. We note that NHEJ-mediated large deletions have been reported in many diseases [48]. In *GPR98*, another large deletion of 136,017 bp had been reported [49] and to our knowledge, the 14,042 bp deletion, reported in this study, is the second large deletion in this gene.

Recently, targeted sequencing approaches aiming at enriching for a subset of Usher genes or related conditions have been used as an alternative to classical methods for molecular diagnosis of Usher syndrome mutations and yielded high rates of mutation detection (70–80%) [17], [50], [51]. Despite the fact that mutation detection rates in these studies cannot be compared with those obtained in our study since some of our patients had linkage to known USH loci, our analysis indicates that WES is equally efficient and yield equivalent mutation detection rate. In addition, WES will allow not only detecting mutations in known genes, but will identify new genes responsible for Usher syndrome in patients who are negative for mutations in the known genes. The high rate of identified novel mutations in our cohort can be attributed to the large sizes of USH genes, their known high rate of private mutations, the lack of previous mutation analysis on Lebanese patients with USH, and the genetic and allelic heterogeneity of USH in Lebanon, which was unexpected because of the small size of the population and its known high rate of consanguinity.

In conclusion, our study highlights the genetic diversity in the causation of USH in the Lebanese population and reiterates the usefulness and efficiency of WES over traditional approaches for molecular diagnosis of USH. Identification of 11 novel causative mutations for USH in Lebanon adds to the existing causative alleles of USH and genotyping these alleles would be helpful as a first screen in diagnosis of USH patients in Lebanon.

## Supporting Information

Figure S1 **Pedigrees of the Usher syndrome type 1 families analyzed in this study.**
(TIF)

Figure S2 **Pedigrees of the Usher syndrome type 2 families analyzed in this study.**
(TIF)

Figure S3 **Chromatograms of the patients with** ***MYO7A*** **,** ***GPR98*** **,** ***USH2A*** **and** ***CDH23*** **mutations.**
(PDF)

Table S1 **Segregation of the identified mutations in the analyzed USH1 families.**
(XLSX)

Table S2 **Segregation of the identified mutations in the analyzed USH2 families.**
(XLSX)

Table S3 **Sequence coverage of the 10 Usher genes in whole exome sequencing.**
(XLSX)
