# Supplementary material for: Molecular Genetics of the Usher Syndrome in Lebanon: Identification of 11 Novel Protein Truncating Mutations by Whole Exome Sequencing
Source: PLoS One. 2014 Sep 11;9(9):e107326. doi: 10.1371/journal.pone.0107326 (PMC4161397; doi:10.1371/journal.pone.0107326)
Supplement: Figure S3 — Chromatograms of the patients with MYO7A , GPR98 , USH2A and CDH23 mutations. (PDF) [file pone.0107326.s003.pdf]

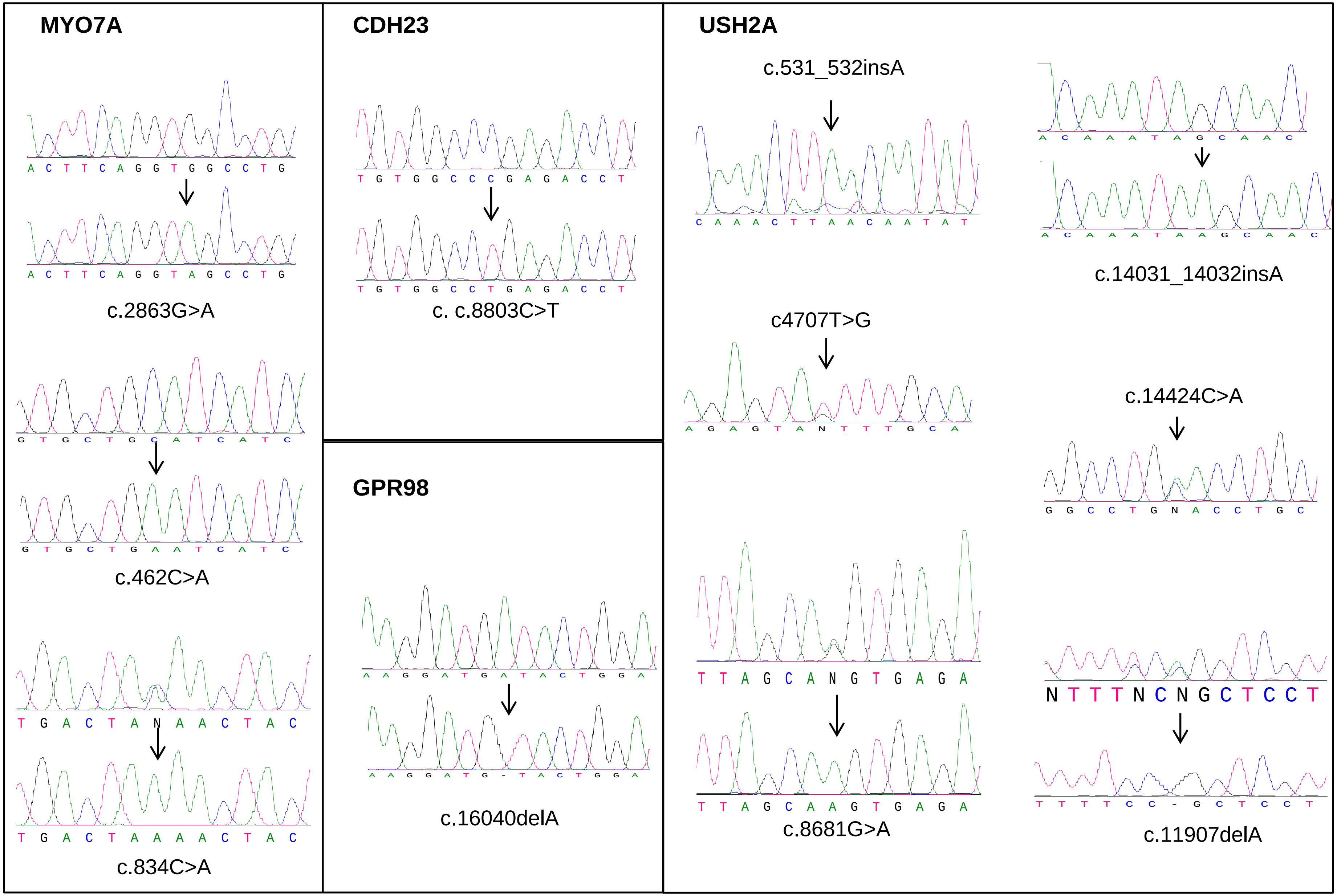

Fig S3: Electrophorograms of the patients with MYO7A, GPR98, USH2A and CDH23 mutations. When available, wild type electrophorograms is above the mutated one and the arrow indicates the changed nucleotide.
